# Alterations of neural activity in the prefrontal cortex associated with deficits in working memory performance

**DOI:** 10.3389/fnbeh.2023.1213435

**Published:** 2023-10-17

**Authors:** Sihai Li, Matthew C. Rosen, Suha Chang, Samuel David, David J. Freedman

**Affiliations:** ^1^Department of Neurobiology, The University of Chicago, Chicago, IL, United States; ^2^Neuroscience Institute, The University of Chicago, Chicago, IL, United States

**Keywords:** working memory, prefrontal cortex, neurophysiology, cognitive deficits, brain stimulation

## Abstract

Working memory (WM), a core cognitive function, enables the temporary holding and manipulation of information in mind to support ongoing behavior. Neurophysiological recordings conducted in nonhuman primates have revealed neural correlates of this process in a network of higher-order cortical regions, particularly the prefrontal cortex (PFC). Here, we review the circuit mechanisms and functional importance of WM-related activity in these areas. Recent neurophysiological data indicates that the absence of these neural correlates at different stages of WM is accompanied by distinct behavioral deficits, which are characteristic of various disease states/normal aging and which we review here. Finally, we discuss emerging evidence of electrical stimulation ameliorating these WM deficits in both humans and non-human primates. These results are important for a basic understanding of the neural mechanisms supporting WM, as well as for translational efforts to developing therapies capable of enhancing healthy WM ability or restoring WM from dysfunction.

## 1. Introduction

Working memory, the ability to hold information in mind and use it to guide ongoing behavior, is a linchpin of cognition (Baddeley, 2012). This faculty is generally indispensable for flexible behaviors. When humans and other animals act, they need to make choices that reflect myriad influences—what their sensory systems indicate about the present state of the world, any of several kinds of goals, features of the environment learned through prior experience, etc. Willful consideration of any of these sources of information requires animals to use their WM. WM encompasses brain systems that can rapidly select from and transform the contents of short-term memory stores to affect behavior. This ability to flexibly control internally held information is critical for many behaviors we execute every day (reading, abstract thought, planning, etc.).

Impairments of this critical ability, frequently observed in nervous system diseases and during aging, are behaviorally debilitating. WM deficits affect various cognitive functions, including learning (Mammarella et al., 2010), planning (Altgassen et al., 2007), comprehension (Savage et al., 2007), etc. Given the widespread impact of WM dysfunction, there is a pressing need to develop effective therapies to address these deficits. However, effective treatments require a deep understanding of the neural mechanisms underlying WM function and dysfunction. The first answers to this basic question about mechanisms underlying WM come from primate neurophysiology: some neurons, especially in frontal and parietal areas, are strongly active specifically while animals hold task-relevant information in mind. These observations formed the core of a common framework for WM’s neural mechanism (“persistent activity”), in which elevated, memory-specific activity bridging stimulus presentation and the behavioral response continually and actively maintains the contents of WM (Funahashi et al., 1989; Wang, 2021). Complementary evidence has since broadened this perspective to include additional physical substrates for active maintenance without spiking (Miller et al., 2018; Lundqvist et al., 2018b) and for maintenance in non-active formats (Stokes et al., 2013; Stokes, 2015; Masse et al., 2019). In light of this proliferation of mechanisms, here we adopt a viewpoint of WM as a union of multiple mechanisms rather than one unitary mechanism. We outline the implications of this multifactorial view for our understanding of how and why WM deficits arise, as well as the nascent field of WM rehabilitation (Beynel et al., 2019; Reinhart and Nguyen, 2019; Grover et al., 2022).

## 2. Neuroanatomy of WM

For clarity, we begin with an explicit definition of WM: when we use the term WM, we refer to a system that actively maintains or manipulates internally held representations of information and uses them to support ongoing behavior (Masse et al., 2019, 2020). In our definition, WM involves but is distinct from “short-term memory,” which we take to comprise the set of functions capable of temporarily maintaining representations of information. This distinction, though important, has recently been comprehensively reviewed (Cowan, 2017), and is thus not a central focus of this review. Henceforth in this review, we will focus primarily on visuospatial WM, the subsystem that represents spatial (and other) relationships among visual stimuli.

### 2.1. The textbook picture of WM localization

The neuroscience of WM began with studies of functional localization—identifying which regions of the brain are important for maintaining and manipulating information in mind. Central to these efforts was the development of the delayed-response task paradigm, a behavioral assay of WM in which subjects are presented briefly with a stimulus and, after a short delay, required to generate a response contingent on the memory of that stimulus. Much of the evidence obtained by early studies using these delayed-response tasks implicated the prefrontal cortex as a critical node for WM: lesions to the PFC impaired humans’ and monkeys’ ability to successfully execute appropriate responses after the delay (Jacobsen, 1935; Milner, 1963; Perret, 1974), and single-neuron extracellular electrophysiology in monkeys found cells in PFC with strikingly elevated selective delay activity when the animals needed to maintain a memory of the earlier stimulus (Fuster and Alexander, 1971; Kubota and Niki, 1971).

These paradigms for studying WM benefited further from importing approaches from sensory neurophysiology–systematic manipulation of sensory features of the stimuli to be held in mind. Goldman-Rakic and others applied this methodology to the study of WM by manipulating the spatial locations of task stimuli; their results confirmed and extended earlier findings supporting PFC as a critical node for WM because they found that some PFC neurons’ activity was not just elevated during the delay, but elevated in a manner specific to the spatial location of the remembered stimulus (Funahashi et al., 1989). Follow-up studies extended findings that PFC activity not only represents spatial stimulus features, but also other non-spatial stimulus features like motion direction (Zaksas and Pasternak, 2006), object (Wilson et al., 1993), prospective code (Rainer et al., 1999), abstract rules (Wallis et al., 2001), categories (Freedman et al., 2001), color (Min et al., 2021), etc. Additionally, this spatially-selective delay activity was later found to extend beyond neurons in the PFC and into other high-order regions related to attentional control, including the posterior parietal cortex (Chafee and Goldman-Rakic, 1998), leading to a view of WM as centered in the PFC, with related representations in high-order regions of the parietal cortex with which the PFC is strongly interconnected.

Despite the observation of memory-specific delay activity localized to PFC and posterior parietal cortex (PPC), interpreting such activity as the substrate of WM information contents suffers from a uniqueness problem: the converse implication (that other areas are not the substrate of information maintenance in WM) is difficult to adjudicate in the absence of whole-brain activity measurements and cannot accommodate a role for subthreshold, non-spiking, mechanisms in information maintenance. These concerns have prompted the development of new theoretical models of WM as a process distributed across several brain regions rather than one localized to the frontal or parietal lobes in particular. Although they differ in their details, these models tend to feature PFC as maintaining and broadcasting executive signals that bias activity in other brain regions, which function approximately as buffers for fine-grained representations of mnemonic content (Miller and Cohen, 2001; Lara and Wallis, 2014, 2015; Christophel et al., 2017; Froudist-Walsh et al., 2021). Overall, PFC is generally thought to carry signals that play a causal role in WM, and which are often correlated with the details of the information held in mind.

### 2.2. Microcircuit mechanisms of WM

Whether elevated delay activity in PFC serves to maintain or to control information held in mind, understanding how it arises remains an open challenge in the neuroscience of WM. A combination of theory and experimentation has identified several key elements of the mechanism: biological processes with relatively long-time constants decouple PFC activity from the offset of sensory stimuli by driving reverberant spiking in recurrently connected neuronal circuits of the PFC. This class of long-time-constant processes includes the activation of NMDA receptors by prolonged elevated presynaptic activity (Lisman et al., 1998), activity-dependent alterations to synaptic dynamics (Wang et al., 2006; Mongillo et al., 2008), and calcium channels with slow inhibition kinetics that hold resting potentials near threshold (Helton et al., 2005), among others.

**Figure 1:**
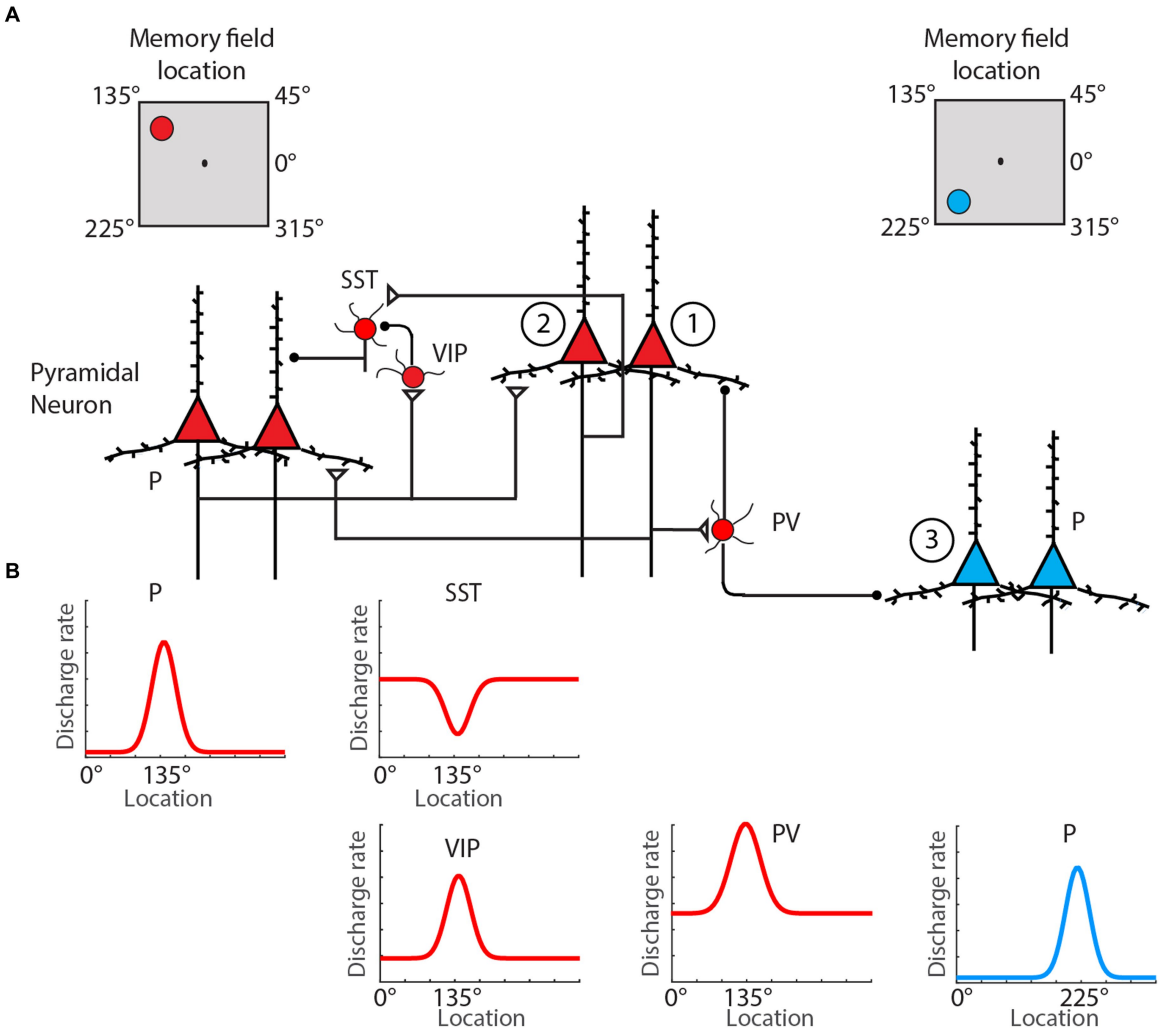
Classical spatial WM microcircuit motif. **(A)** Pyramidal neurons (red triangles) with the same preferred location mutually excite one another directly via the basal dendrites, while also driving inhibition of pyramidal cells with different preferred locations (blue triangles) through excitation of PV interneurons. Selective gating of excitation arriving through pyramidal cells’ apical dendrites is regulation by an inhibition-of-inhibition motif involving VIP and SST-type interneurons. **(B)** Schematic of firing rate by cell type as a function of spatial location during spatial WM delay. Neuron types: pyramidal (P), parvalbumin (PV), somatostatin-expressing (SST), vasoactive intestinal polypeptide-expression (VIP). Adapted from Li et al. (2020).

These molecular mechanisms are complemented by circuit interactions, both within and outside the PFC, that sustain delay activity. The basic conceptual model holds that the presentation of a stimulus to be remembered triggers an initial excitatory drive that kindles activity in a subnetwork of PFC neurons, where the identity of the subnetwork reflects the identity of the stimulus (Li et al., 2020; Figure 1). After the offset of the stimulus, this subnetwork’s activity remains elevated until the engagement of a stimulus-dependent behavioral response. Early theories suggested that this elevated activity reflected strong connections among the excitatory neurons within the relevant PFC subnetwork (Compte et al., 2000; Wang, 2001). These recurrent loops are now also known to extend outside the PFC—in some settings involving cortico-thalamocortical loops (Halassa and Kastner, 2017)—and to involve several inhibitory interneuron subtypes. While recurrent connections among excitatory neurons help to maintain stimulus-driven activity, dendrite-targeting (SOM) interneurons make this activity robust by selectively weakening irrelevant incoming projections. Conversely, relevant information can modulate the active PFC subnetwork via the disengagement of specific subsets of SOM interneurons, an effect modulated by inhibitor-of-inhibitor VIP neurons (Yang et al., 2016). Recurrent networks in the PFC also manifest synaptic specializations that promote persistently elevated levels of activity (Wang et al., 2006). In particular, recurrent connections among some subnetworks of pyramidal cells in the PFC are enriched relative to those observed in primary sensory cortical regions and are more frequently facilitating (Wang et al., 2006).

## 3. Neural substrates of WM: a broadening view

Early neurophysiological research on nonhuman primates demonstrated that neurons in the PFC not only responded to sensory stimuli, but also remained active after the stimuli were no longer present. This research provided evidence that a neural correlate of WM was “persistent activity” (Fuster and Alexander, 1971; Funahashi et al., 1989). Subsequent studies have shown that persistent activity in the PFC predicts successful WM recall and the accuracy of behavioral responses in visuospatial WM tasks (Zhou et al., 2013; Li et al., 2021). Computational models based on the concept of persistent activity have also advanced our understanding of WM’s neural mechanisms and replicated experimental findings (Wimmer et al., 2014; Xie et al., 2022). However, alternative models of WM have begun to extend the mechanisms that are recognized to potentially contribute to short-term memory, including WM. These models fall into two categories: models based on rhythmic activity (Miller et al., 2018; Lundqvist et al., 2018b) and models without changes in mean firing rate, known as “activity-silent” models (Stokes et al., 2013; Stokes, 2015; Masse et al., 2019). In this review, we discuss these different WM models and their potential in explaining mechanisms underlying WM.

### 3.1. Persistent spiking activity and WM representation

**Figure 2:**
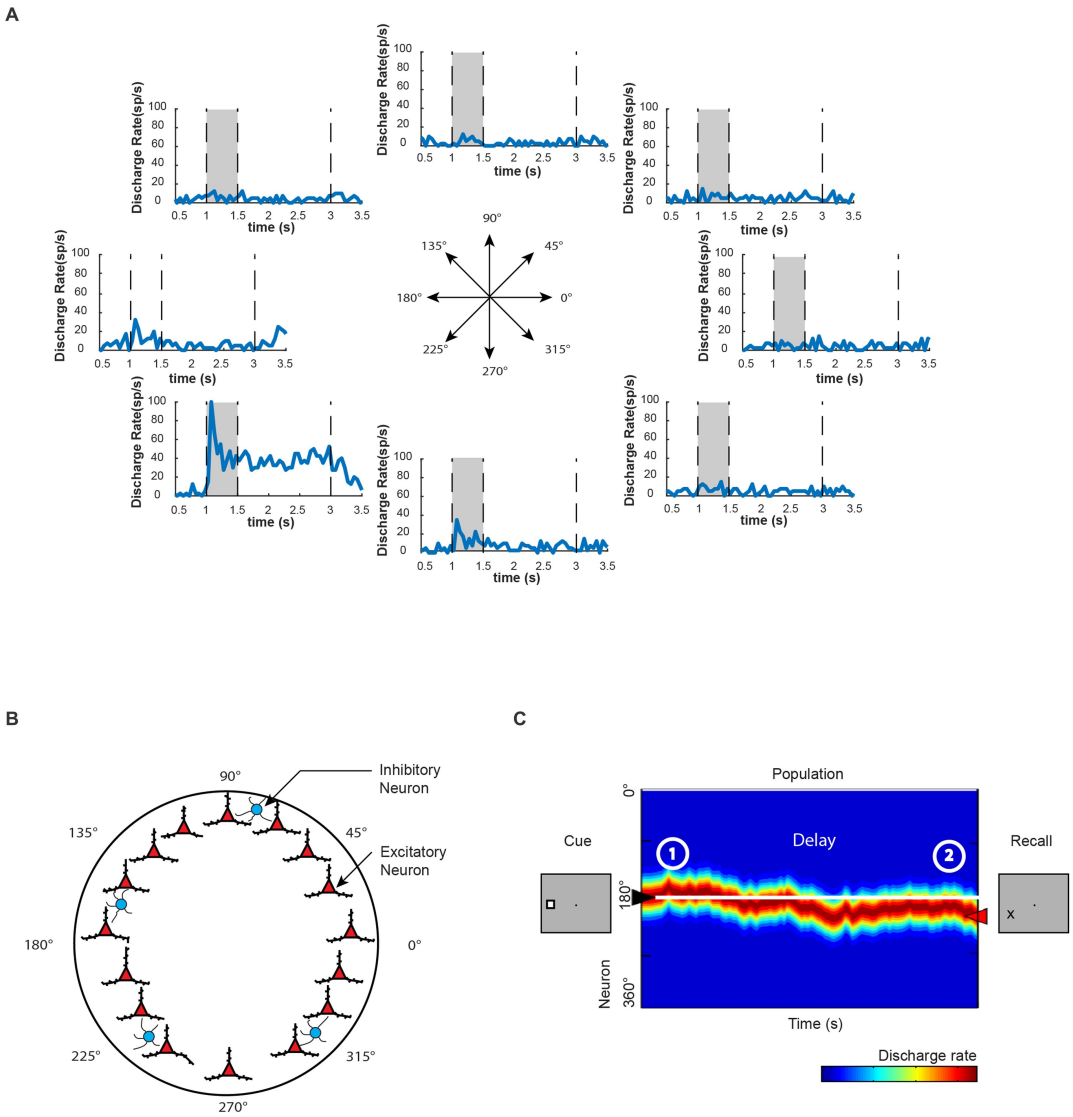
Persistent activity of WM. **(A)** Neuronal activity of an example prefrontal neuron during the execution of the oculomotor delayed response task. During this task, a visual cue is presented to the monkey within a specific area of its visual field, after which the cue disappears. The monkey must then remember the location of the visual cue during a delay period before responding to the task. Once the monkey is signaled, it makes an eye movement to the remembered location of the visual cue. This particular neuron exhibits the most selective activation during the presentation of the visual cue located at 225° relative to the horizon. It also maintains persistent activity during the delay period in a spatially selective manner. **(B)** Computational model proposes possible mechanisms underlying WM representation. Here, information can be code in a cell-specific manner in excitatory neurons (red triangles), and each neuron behaves similar to the example neuron in A. Inhibitory cells are symbolized by blue circles. Adapted from Constantinidis and Klingberg (2016). **(C)** Schematic diagram of population neural activity in the neural network shown in B, with colors indicating the level of activity (blue: low activity; red: high activity). The peak of neural network activity indicates the remembered location in WM. In this example, the neural network successfully maintains the WM information, although there are occasional fluctuations in activity levels. Adapted from Li et al. (2021).

Spatial information representation in WM is a longstanding topic of study in systems and cognitive neuroscience. Visuospatial WM has been the focus of pioneering research because the spatial locations of sensory stimuli can be parametrically manipulated and thus allow for systematic measurement of corresponding neural activity. Classic visuospatial WM tasks, such as the oculomotor delayed-response task (ODR), provide an excellent platform for investigating WM’s neural mechanism (Watanabe et al., 2009). Neurophysiological recordings from nonhuman primates have revealed individual neurons exhibiting persistent activity with visual stimulus selectivity for different spatial locations during the task (Figure 2A) and a proportion of neurons in the dorsolateral prefrontal cortex (dlPFC) are particularly specialized for representation of spatial location. The information of remembered visual stimuli location could be further decoded from the neuronal population’s activity (Meyers et al., 2008, 2012).

Computational studies considering mechanisms of persistent activity have played a significant role in advancing our understanding of the neural mechanisms underlying spatial WM. Biophysically realistic models have been developed to simulate persistent activity through a population of neurons with similar spatial tuning properties, in which activity reverberates (Compte et al., 2000; Figure 2B). Such network models can exhibit a bell-shaped pattern of activity, also known as a bump, even without external input during delay period of a WM task that is specifically tuned to produce this pattern (Figure 2C). Due to the network structure, neural activity would be naturally attracted toward the bump state. Thus, this network is referred to as a “bump attractor.” The “bump attractor” models have yielded insights into the predictive relationships between the variability of prefrontal activity during the delay period and the fine details of recalled spatial location, as observed in neurophysiological results (Wimmer et al., 2014). Each node in the network represents a neuron with the peak of its receptive field at a different location, forming a ring of neurons equidistant from the fovea. The strength of connectivity between neurons in these models is dependent on the distance of their preferred receptive field locations. This architecture allows for the maintenance of afferent input through recurrent excitations, and the peak of population activity in the network represents the information of the remembered location, behaving as a continuous attractor (Figure 2C). Simulation results from the bump attractor model networks indicate that drifts in population neural activity are tied to deviations in behavioral outputs. Results from a match-to-sample WM task further support the model’s predictions that the peak of PFC population activity represents the contents of WM, and abnormal neuronal activity during the delay period, deviating from the average firing rates in correct trials, is correlated with incorrect behavioral output, as predicted by the bump attractor model (Li et al., 2021). Earlier studies suggested that error trials in similar WM paradigms were only due to significantly lower or even vanishing delay period activity (Funahashi et al., 1989; Zhou et al., 2013). However, recent bump attractor models and a series of neurophysiological studies suggest that despite the existence of persistent activity during the delay period, the drift of populational activity representing inaccurate WM information can still lead to error responses.

Earlier WM studies primarily focused on the representation of spatial information, while more recent research has expanded to include a broader range of information, such as stimulus features and internal state variables. Several reports find persistent activity in PFC that represents various sensory features during WM, including objects and natural images (Miller et al., 1996), luminance (Constantinidis et al., 2001), color (Buschman et al., 2011), categories (Freedman et al., 2001; Parker et al., 2002; Roy et al., 2010), and motion (Mendoza-Halliday et al., 2014; Masse et al., 2017). In addition to representing stimulus characteristics, PFC activity can also persistently encode abstract task rules (Wallis et al., 2001; Mansouri et al., 2020), item quantity (Nieder et al., 2002), and reward expectations (Leon and Shadlen, 1999).

However, whether persistent activity encoding the information held in mind is sufficient for WM representations remains unresolved. A recent debate (Constantinidis and Klinberg 2016; Lundqvist et al., 2018a) has suggested that persistent activity may sometimes reflect an artifact of analyses based on trial- and time-averaging of neural activity. Although trial-averaged spiking can facilitate some kinds of statistical analysis, averaging spikes across epochs of the trial and across different trials can obscure details of WM-related neuronal spiking on single trials (Miller et al., 2018; Lundqvist et al., 2018a,b). Contrarily, Lundqvist et al. proposed a shift in focus for future studies toward local network activity, rather than individual neuronal spiking. Their suggestion stems from the notion that WM maintenance appears to encompass sparse, transient coordinated activations of local networks, rather than continuous, asynchronous persistent neuronal activity. In fact, observations indicate that the activity on individual trials often displays sparsity beyond what average persistent activity might imply. And between spikes, memories are carried by temporary changes in synaptic weights, which is also an important aspect of WM representation and should not be neglected. Although attractor models in theory explain some key features of WM—in particular, its limited capacity–future work must determine whether they also can do so under biological constraints, e.g., of metabolic constraints (Attwell and Laughlin, 2001; Masse et al., 2019). We believe that these persistent uncertainties reflect the need to establish more precisely what computational role persistent activity plays in WM—maintaining information itself, controlling another short-term memory buffer, or some other function entirely.

### 3.2. Local field potential rhythms and the control of WM

The same kind of experimental paradigm and measurement that identified persistent activity as neural substrate of WM also yielded an important addition to the understanding of WM mechanism: oscillations in the local field potential (LFP) in PFC (Siegel et al., 2009; Kornblith et al., 2016; Lundqvist et al., 2016). The encoding of information may also depend on the temporal dynamics between neurons, specifically, the precise phase alignment of spikes relative to the rhythmic activity across the neuronal population. When decomposed by frequency, these oscillations show modulations of power in specific bands that co-occur with the animal controlling information in its WM. Bursts of power in the gamma frequency band (50–120 Hz) are coupled to spiking that carries information about remembered WM information. During these gamma bursts, spiking was more elevated when the contents of WM were more informative, and little gamma band power was recorded from sites where spiking did not carry any WM information. In contrast, beta bursts (~20–35 Hz) display an opposite relationship, relating instead to the suppression of informative spiking and WM reallocation. During the post-trial period of a WM task that no longer required animals to maintain central fixation and WM information, elevated beta bursting was reported from informative sites, but not from non-informative sites. An interactive balance between beta and gamma bursting has been suggested to correlate with WM control, and the amplitude of gamma and beta bursts was found to be anti-correlated. While animals retained information in their WM to guide an upcoming report, gamma bursting increased and beta decreased; when this information needed to be cleared, beta bursting increased and gamma bursting decreased (Lundqvist et al., 2018b). These observations suggest that WM can engage both sparse and sustained spiking mechanisms, and further that the emergence of such activity may be gated by gamma and beta-band oscillations of the LFP.

Our understanding of LFP oscillations’ role in controlling WM is still in its infancy and there are several concerns regarding the current understanding of how LFP rhythms underlies and modulates WM. Rhythmic models often focus on oscillatory activity which is postulated to gate access to WM and clear out WM information after use (Bastos et al., 2018), but these types of control mechanisms raise several questions. First, the ability to maintain stable information over a short period of time is essential for WM, but internal state changes can cause slow drifts in PFC neuronal activity that can lead to behavioral fluctuations (Cowley et al., 2020). When simulated in attractor models, such unstable activity during the delay period can lead to WM representation fluctuations and can predict deviant behavioral responses (Wimmer et al., 2014; Li et al., 2021). However, it is still unclear whether and how such deviations in behavioral responses and underlying neuronal activity correlate with changes in gamma bursting. Although one study reported differences in gamma bursting between correct and error trials in WM tasks (Lundqvist et al., 2018b), significant differences in gamma bursting were only identified during the period when testing stimuli were presented, not during the delay period that is most relevant for WM maintenance. Future studies can work on developing perturbation approaches which can specifically drive changes in local field potential oscillations to test the involvement of LFP modulating WM. Even within established WM paradigms like the ODR task, it will be valuable to investigate whether and how gamma bursting relates to trial-to-trial behavioral variability. This has potential to enhance our understanding of the relevance of LFP oscillations to working memory processes.

Another observation is that the frequency range associated with gamma bursting has varied in different studies, with reported ranges including 50–120 Hz (Lundqvist et al., 2018b), 30–80 and 80–500 Hz (Jia et al., 2017), and 25–90 Hz (Pesaran et al., 2002). Such variability has led to a circumstance that some low gamma frequencies in one study correspond to the range of beta frequencies in another study, which is contradictory since beta frequencies are thought to be related to the suppression of object information in spiking (Lundqvist et al., 2018b). Thus, although oscillatory activity provides a continuous measure of WM-related neural signals compared to sparse activity in single-unit analysis, the conflicting nomenclature of frequency ranges in different studies raises a question about the most relevant frequency bands for WM and whether the functionality of these bands varies across different WM paradigms. Furthermore, it is unclear how different frequency bands map onto distinct components of WM processing.

Importantly, persistent spiking activity and LFP oscillations may reflect similar aspects of WM. For instance, the magnitude of persistent activity during the delay period of a WM task increased monotonically as a function of number of remembered stimuli during the delay period (Tang et al., 2019). Correspondingly, it has been noted that increased gamma frequency power corresponds to the increased number of memorized visual stimuli in a WM task, indicative of heightened WM loads (Lundqvist et al., 2016). Thus, it is not accurate to view them as conflicting mechanisms for WM representation. The canonical model proposed by Goldman-Rakic (1995), which was later formalized as a computational model (Compte et al., 2000), did not specify that WM representations were stored solely by the persistent activity of single neurons, but by a population of neurons with recurrent connections. As LFP signals represent the aggregate activity of small populations of neurons by their extracellular potentials, it is plausible that LFP signals reflect the activity of local WM networks, which also demonstrated persistent spiking activities. Therefore, it is important for future studies to investigate the interplay between persistent spiking activity and LFP oscillations, and to explore their complementary roles in WM.

### 3.3. Dynamic mechanisms for maintaining information

Mechanisms that do not rely on sustained encoding via spiking, in particular short-term synaptic plasticity (STP), have also begun to emerge as complementary mechanisms for maintaining information in short term memory. STP belongs to a class of activity-inducible processes that modify synaptic, and by extension, neural circuits’ I/O properties over timescales of several seconds. During STP, this is thought to reflect presynaptic calcium accumulation due to vigorous spiking activity, which can lead to a temporary but slowly-decaying facilitation or depression of release. The post-synaptic effect of spiking in a pre-synaptic cell undergoing STP is therefore history-dependent, reflecting both ongoing membrane potential changes and the recent history of presynaptic calcium entry. In cells with stimulus-specific spiking during WM encoding, this buffering will be stimulus-specific, as will the eventual post-synaptic effect. Through this basic logic, laid out initially by Mongillo et al. (2008), presynaptic residual calcium is proposed to act as a buffer that is loaded and refreshed by spiking activity. Computational studies have also shown that the limited capacity of remembered items, a key feature of WM, can be estimated by the ratio of the characteristic time of short-term synaptic depression to the synaptic current time constant (Mi et al., 2017).

Information maintenance has also been shown to correspond with neural codes that are dynamic and do not involve changes in mean firing rate, in addition to more traditional sustained codes (Stokes et al., 2013)—particularly in tasks requiring more active manipulation of remembered information (Masse et al., 2019). The critical difference relative to more traditional attractor models is thus that the modes of population activity that contain information about WM contents change. Moreover, recent studies have shown that both the activity silent model and classic persistent activity model of WM can coexist and switch as needed (Barbosa et al., 2020; Froudist-Walsh et al., 2021). For instance, during the ODR WM task, Barbosa et al. observed that stimulus information remained present in activity-silent traces between trials and was even decodable before the onset of the consecutive trial. The coexistence of these two models suggests that they can be complementary rather than conflicting, which urges the significance of exploring the interaction between different WM models.

## 4. Impairment of WM in aging, cognitive dysfunction, and disease

Working memory performance can be influenced by a multitude of factors such as aging (Wang et al., 2011), side effects of pharmacological therapy (Reilly et al., 2006; Roussy et al., 2021), and neurodegenerative diseases (Baddeley et al., 1991; Owen et al., 1997; Martinussen et al., 2005). Although these factors can lead to similar WM-related behavioral deficits, the underlying mechanisms of WM impairments may differ. Here we examine recent evidence on how aging, side effects of clinical drug use, and neurodegenerative diseases lead to structural changes in PFC. And as instructed by the previous studies regarding the crucial involvement of PFC neurons in WM functionality, we aim to investigate how alterations in PFC neural activity resulting from aging, clinical drug use, and neurodegenerative diseases impair WM performance by disrupting the neural representation of information.

### 4.1. Aging and WM

There is ample evidence that WM performance declines with age (Hedden and Gabrieli, 2004; Gazzaley et al., 2005). Compared to younger participants, older participants typically perform more poorly in WM tasks. Multiple cross-sectional studies have shown that aging in healthy adults is associated with smaller brains and thinner cortices. One of the brain regions that significantly shrinks over time, along with many other regions, is the PFC (Raz et al., 2010). Imaging studies have also revealed that lower WM performance is associated with decreased cortical surface area in frontal lobe regions among elderly people (Nissim et al., 2017). In their study, healthy elderly individuals with an average age of 70 performed an n-back WM task, followed by an MRI scanning session. N-back performance data were used to classify participants into high and low-WM groups. Their results showed that low WM performers had significantly less surface area in the inferior frontal gyrus, the superior frontal gyrus, and the medial orbital frontal gyrus compared to high WM performers. Notably, decreases in cortical thickness typically indicate neurodegenerative tissue loss (Fischl and Dale, 2000); however, the study did not find any significant differences in cortical thickness between the high and low-WM groups, ruling out neurodegenerative tissue loss as a possible explanation for the observed differences. This study concluded that the decreased cortical surface area in frontal lobe regions induced by aging leads to poor WM performance in the normal aging participants, without the presence of tissue loss induced by neurodegenerative disease.

Although there is evidence that WM performance declines with age, studies in nonhuman primates have shown that the number of neurons in area 46 does not significantly change with age (Smith et al., 2004). Therefore, researchers have focused on investigating changes in synapses to understand the neurobiological basis of age-related WM impairment. In aged monkeys, signs of dendritic degeneration have been observed in some of the dendrites in the upper layers of area 46, particularly in layer 1 (Peters et al., 1994). Furthermore, electron microscopic analysis has revealed an overall loss of approximately 30% of synapses in layers 2/3 in aged monkeys (Peters et al., 2008). Moreover, WM-related persistent activity is thought to be driven by recurrently connected networks of pyramidal cells in layer 3 (Goldman-Rakic, 1995). Consequently, age-related synaptic loss might lead to compromised WM performance. Furthermore, research has shown that aged monkeys experience degeneration of their myelin sheaths, and this leads to an age-related decline in myelinated nerve fibers (20%–40%) in area 46, which has been associated with the disconnection of the PFC from other central nervous system components. The changes in myelin sheaths result in reduced conduction velocity, which in turn affects the timing of neuronal circuits. Additionally, regression analyses have demonstrated that these myelin defects correlate with impaired WM performance (Luebke et al., 2010).

Several electrophysiological and pharmacological studies have shown that aging alters the physiological properties of PFC neurons during WM tasks in aged monkeys (Wang et al., 2011). Neurophysiological recordings on nonhuman primates have revealed that a proportion of recorded PFC neurons showing persistent activation lasting through the cue and delay period of a memory-guided delayed response task, respectively. The sustained activation of these neurons is modulated by the spatial location of visual stimuli and reaches peak responses for the neuron’s preferred location (Goldman-Rakic, 1995). Neurophysiological recordings have demonstrated an age-related decline in the firing rates of neurons showing persistent responses during the delay period, while the firing of neurons actively responding during cue period remains unchanged between different age groups (Wang et al., 2011). Immunoelectron microscopy studies have demonstrated that age-related disinhibition of cyclic adenosine monophosphate (cAMP) signaling leads to a decrease in the persistent activity of area 46 neurons (Arnsten et al., 2010). Remarkably, inhibiting cAMP signaling has been shown to partially restore the memory-related firing activity of neurons that exhibit persistent activity during the delay period of WM paradigms. This discovery suggests that age-related WM deficits may be ameliorated, at least in part, by pharmacologically restoring persistent activity in the PFC (Wang et al., 2011). Altogether, understanding how age-related structural changes in the PFC lead to WM deficits provides insights for developing potential pharmacological and brain stimulation methods to enhance WM performance in elderly individuals.

### 4.2. Double-edged pharmacological effects and WM

WM can be adversely affected by certain medications commonly prescribed to treat behavioral disorders or used as dissociative anesthetics. Benzodiazepines, one of the most widely prescribed medications for anxiety disorders, can lead to various negative effects, including impairments in WM (Guina and Merrill, 2018). Similarly, ketamine, which has been used for pain management, anti-depression, and anti-inflammation for almost half a century, has been shown to induce behavioral abnormalities similar to cognitive WM deficits observed in schizophrenia. Additionally, studies have demonstrated that even administering a subanesthetic dose of ketamine can hinder the monkeys’ performance in executing a rule-based working memory task. This task necessitates subjects to respond based on a cue presented at the start of the trial, signifying whether that trial requires a prosaccade or antisaccade rule (Skoblenick and Everling, 2012; Ma et al., 2015). Here we discuss the latest neurophysiological evidence underlying the effects of these medications on WM ability, and the utility of pharmacological manipulations in gaining understanding cellular mechanisms underlying WM.

Benzodiazepines, gamma-aminobutyric acid (GABA) receptor agonists, are a class of psychoactive drugs that are commonly prescribed to treat anxiety, insomnia, and other related disorders (Shader and Greenblatt, 1993). While benzodiazepines provide unique medical value, they also come with side effects, including the impairment of WM. Clinical studies have shown that long-term benzodiazepine administration can impair WM (Kishi et al., 2010; Fond et al., 2018). This effect likely stems from this drug’s impact on GABAergic signaling. GABA is an inhibitory neurotransmitter that is widely distributed throughout the central nervous system, including in brain regions critical for WM, such as PFC. The importance of GABA in WM is thought to lie in its ability to modulate neural activity and improve the efficiency of WM processes (Yoon et al., 2016). Overdose of benzodiazepines may disrupt this balance by over enhancing GABAergic activity. The PFC contains high concentrations of GABA receptors, and excessive activation of these receptors can lead to over-inhibition in the local circuits and reduce neuronal activity in this region, which can impair the ability of maintaining and manipulating information in WM (Constantinidis and Wang, 2004).

Ketamine, a dissociative anesthetic that has been used for decades in clinical settings to induce anesthesia and sedation, has also gained interest as a rapid-acting antidepressant for treatment-resistant depression (Krystal et al., 2019). While ketamine has therapeutic benefits, it is also known to impair WM in both humans and animals even under subanesthetic doses (Taffe et al., 2002; Morgan and Curran, 2006). This impairment occurs because ketamine blocks the N-methyl-D-aspartate (NMDA) receptors in the brain; these receptors are involved in the regulation of synaptic plasticity, learning, and memory (Kapur and Seeman, 2002; Zorumski et al., 2016). The blockade of these receptors also leads to a decrease in glutamate transmission, which in turn affects the activity of other neurotransmitters, such as dopamine and acetylcholine, both of which are involved in WM (Brozoski et al., 1979; Verma and Moghaddam, 1996; Narendran et al., 2005). Studies have shown that acute administration of ketamine in humans impairs WM performance in tasks that require attention and WM-based executive function, such as the n-back task (Morgan et al., 2010; Abdallah et al., 2017).

In addition to acute effects, chronic ketamine use has also been associated with WM deficits. A study in rats found that chronic ketamine administration reduced the number of NMDA receptors in the prefrontal cortex (Xu and Lipsky, 2015). This reduction in NMDA receptors is believed to contribute to the long-term impairment of WM observed in chronic ketamine users. A neurophysiological study in nonhuman primates also revealed that the administration of ketamine under subanesthetic dose lowered activities of neurons showing elevated activities during delay period of WM tasks and also reduced the spatial tuning (Wang et al., 2013). PFC neuronal ensembles can encode spatial WM, but ketamine disrupts this encoding (Roussy et al., 2021). Notably, their findings indicate that the administration of ketamine did not impair performance during the perception process, suggesting that the ketamine-induced deficit specifically affected the WM process. Thus, it is important to consider the potential cognitive side effects when using this drug in a clinical setting, even under a subanesthetic dose, especially when it comes to WM.

### 4.3. Neurodegenerative disease and WM

Working memory impairments are also prevalent in clinical cases of multiple neurodegenerative disorders. Common neurodegenerative diseases such as Alzheimer’s disease (AD) and Parkinson’s disease (PD) are shown to result in WM deficits, and the related symptoms of WM deficits are crucial for early diagnosis of these disorders. Studies relating these diseases’ pathology to this executive dysfunction have also shed light on how mechanisms like oscillatory patterns or persistent activity are involved in WM processes.

Memory loss is a key feature of Alzheimer’s disease, and deficits in working memory can be presented in the preclinical or early stages of AD (Huntley and Howard, 2010; Garcia-Alvarez et al., 2019). Longitudinal studies have shown that compared to older individuals who may experience normal age-related cognitive decline, AD patients’ WM performance deteriorates more rapidly over time (Baddeley et al., 1991; Johnson et al., 2009). Researchers have investigated how AD pathologies such as tau neurofibrillary tangles (NFTs) and beta-amyloid (Aβ) plaques may accelerate cognitive dysfunction in brain regions that support WM. The accumulation of NFTs is associated with global cognitive decline in both AD patients and elderly adults (Arriagada et al., 1992; Schöll et al., 2016), but studies have suggested that the additional presence of Aβ plaques can increase NFT formation and facilitate their spread to more neocortical areas like in frontal cortex (Pooler et al., 2015; Wang et al., 2016; He et al., 2018). Decreased performance on tasks testing for executive function has also been correlated with increased NFTs in frontoparietal regions (Bejanin et al., 2017).

Moreover, a multimodal study that tracked AD biomarkers with PET imaging while recording MEG signals from patients found that increased tau accumulation and global cognitive deficits were correlated with reduced resting-state synchrony in the alpha band (Ranasinghe et al., 2020). Synchronous oscillations are hypothesized to mediate inter-areal functional communication that helps maintain contents of WM (Uhlhaas and Singer, 2006; Rezayat et al., 2022). In healthy subjects, synchrony in the alpha and also the beta band has been found to increase with working memory load or reflect WM contents, especially across frontoparietal networks (Palva et al., 2010; Salazar et al., 2012). However, AD subjects have demonstrated reduced alpha or beta synchrony, both during resting-state studies (Koenig et al., 2005; Stam et al., 2005; Smailovic et al., 2018) and active performance of WM tasks (Pijnenburg et al., 2004). Such abnormalities could signal a functional disconnection in brain activity that impacts maintenance of WM. Future electrophysiological studies in clinical populations could not only illuminate not only the role of synchronous oscillations in WM processes but also improve non-invasive metrics for AD diagnosis.

In Parkinson’s disease, patients also exhibit cognitive impairments in addition to its widely known motor symptoms. Individuals with PD show deficits across visuospatial working memory tests that manifest even in early disease states (Owen et al., 1997; Siegert et al., 2008). Since PD pathology is primarily characterized by the degeneration of dopaminergic neurons in midbrain structures, it is widely hypothesized that cognitive dysfunction stems from a resulting disruption of dopaminergic signaling involving frontal areas that are connected to these subcortical areas through frontostriatal circuits (Owen et al., 1998; Sawamoto et al., 2008) or mesocortical pathways (Mattay et al., 2002). Aforementioned, PFC plays a crucial role in maintaining WM information and it particularly contains a large concentration of D1 dopamine receptors (Lidow et al., 1991). For instance, dopamine at D1 receptors has been proposed to modulate glutamatergic signaling at presynaptic sites in local pyramidal neurons within prefrontal circuits to help control recurrent excitation (Gao et al., 2001). This dopamine-mediated mechanism of recurrent excitation is highly relevant to persistent-activity based models of WM, and this function of WM has been found to be disrupted if dopamine levels are lowered in the PFC. Pharmacological manipulations in non-human primates have shown that dopamine depletion in PFC impairs subjects’ WM performance on a delayed response task (Brozoski et al., 1979), and Sawaguchi also showed that applying dopamine antagonists to D1-receptors in dlPFC significantly reduced dlPFC neurons’ location-specific delay period activity (Sawaguchi, 2000).

## 5. Interventional approaches for enhancing WM

As discussed so far, research from multiple domains has given important insight into the neural mechanisms underlying WM, and has identified factors that can contribute to impairment of WM. Recent work has also started to identify approaches for enhancing, and potentially restoring, WM through both invasive and non-invasive means (Reinhart and Nguyen, 2019; Subramaniam et al., 2021).

In recent years, functional neuromodulation has offered therapeutic benefits through deep brain stimulation (DBS) for a variety of neurological and psychiatric disorders. DBS offers the potential to stimulate or suppress the functioning of neural populations within a radius of a stimulating electrode. Electrode leads are implanted in the target neuronal structure, electrical pulses are applied to modulate neural responses near the lead and alter the neuronal activity to rectify abnormal neural function. DBS has been accepted as an invasive procedure for alleviating cognitive deficits in patients with Parkinson’s disease (Fasano et al., 2012; Okun, 2012) and Alzheimer’s disease (Luo et al., 2021). In one study, Merkl and colleagues investigated the effects of bilateral subthalamic nucleus DBS on the WM of Parkinson’s patients using an n-back task. They found that patients reported feeling more alert and less sedated during short-term DBS-ON periods, and they also demonstrated improved accuracy in the WM task (Merkl et al., 2017).

Similar results have been reported in DBS research in non-human primates, where a series of studies targeted the nucleus basalis (NB) of Meynert, a key structure associated with attention control and arousal—two key functions for learning and memory (Liu et al., 2017). The results showed a clear improvement in cognitive performance with DBS; intermittent stimulation led to the most beneficial effects on WM performance, while continuous stimulation resulted in impaired performance. Furthermore, simultaneous stimulation of the nucleus basalis and recordings in downstream target areas such as PFC during a memory-guided saccade task revealed distinct patterns of neural activity responsible for the improvement in WM stability (Qi et al., 2021). The study reported increased PFC activity during the delay period of a spatial WM task and broadening of each neuron’s selectivity for stimuli with NB stimulation. A significant decrease of Fano factor, a measure of trial-to-trial variability, was observed under NB stimulation, which indicates that NB stimulation made the local network maintaining information in memory more stable.

Emerging non-invasive stimulation techniques provide a safer and more adaptable tool than invasive stimulation in both research and clinical settings. Non-invasive brain stimulation refers to a group of techniques that can modulate brain activity without the need for invasive procedures such as surgery and they are becoming an increasingly common tool to study and treat a range of neurological and psychiatric disorders. These techniques include transcranial magnetic stimulation (TMS), transcranial direct current stimulation (tDCS), and transcranial alternating current stimulation (tACS), and they have been shown to be effective tools to enhance cognitive functions (Begemann et al., 2020). For instance, Meng and colleagues applied TMS, a non-invasive technique that uses magnetic fields to electrically stimulate a targeted area of the brain, to elderly individuals with subjective cognitive decline and found that it enhanced WM performance and significantly improved attention and executive function. The improvement of WM performance was accompanied by recovery in the amplitude of event-related potentials (Liu et al., 2021). Thus, Meng and colleagues speculated that TMS enhanced visual WM performance in elderly individuals by inducing a recovery of event-related potentials. Meanwhile, tDCS works by delivering electrical current through electrodes placed on the scalp, which can potentially modulate the excitability of neurons in underlying brain regions and thereby affect cognitive processes such as WM (Stagg and Nitsche, 2011; Zhao and Woodman, 2021). In one study, patients with Parkinson’s disease showed a significant improvement in task accuracy in WM after active anodal tDCS of the left dorsolateral prefrontal cortex (Boggio et al., 2006). tACS, which is similar to tDCS but involves the delivery of a time-varying rather than constant electrical current through scalp electrodes, and has also resulted in lasting improvements to WM (Reinhart, 2017). Some of this effect is thought to reflect stimulation reinstating cross-frequency coupling between theta and gamma-band oscillations. This coupling has been proposed to constitute a flexible mechanism for combining information across different temporal scales within local cortical networks, and is a prominent feature of WM representation (Axmacher et al., 2010). In physiological states associated with impaired WM, however, including old age, cross-frequency coupling is notably diminished. A recent study by Reinhart and Nguyen investigated the effects of tACS on both WM performance and cross-frequency coupling (Reinhart and Nguyen, 2019). After a period of tACS stimulation over prefrontal cortex, older adults showed a significant increase in WM task accuracy relative to performance changes from sham stimulation. EEG data was recorded immediately after tACS administration, and the subjects exhibited significant changes in the observed theta and gamma amplitudes. Stimulation had restructured theta–gamma coupling such that the strength of this coupling became significantly predictive of individual working-memory accuracy. This rapid improvement in WM performance, including significantly better task accuracy and faster reaction time in the WM task in these subjects which outlasted the stimulation epoch in a 50-min post-stimulation period. In a recent study, the same researchers devised a novel repetitive tACS protocol that modulated theta rhythms in the parietal cortex, leading to improved WM performance. The bipolar sinusoidal current was applied at 4 Hz and demonstrated lasting effects that persisted for days or even a month after intervention (Grover et al., 2022).

These promising findings suggest the potential for non-pharmacological and non-invasive interventions to effectively treat cognitive decline affecting WM. However, it is important to note that specific location and frequency combinations of the stimulation are critical for achieving optimal effects on WM improvement. Previous studies have shown that both theta and gamma frequency rhythms contribute to WM function (Axmacher et al., 2010), but gamma modulation of the parietal cortex did not improve WM performance. This raises questions about the neural mechanisms of non-invasive stimulation in modulating WM ability and underscores the challenge of designing effective stimulation plans to achieve optimal outcomes.

## 6. Conclusion and open questions

This review provides a summary of recent research, debates, and insights into mechanisms underlying the maintenance and manipulation of information in WM. Together, this recent work suggests that although WM relies on a broad, distributed brain network, the unique cellular and circuit organization of the prefrontal cortex is crucial for WM. Current theories of persistent activity models, rhythmic models, and activity silent models offer potential mechanistic explanations of WM, with each model replicating some of the neurophysiological findings from research in humans and animal models. While WM is essential for many cognitive functions, several factors can impair WM performance. However, advancements in brain stimulation techniques have shown promise in improving WM performance in patients with cognitive decline. Brain stimulation interventions not only improve WM performance during the intervention period or a short period after the intervention, but also show promise for providing long-lasting WM improvement for days or even weeks after the intervention.

Many questions remain unanswered regarding the mechanisms underlying WM, and regarding the effectiveness of brain stimulation techniques in improving WM. Firstly, there is ample neurophysiological evidence supporting the role of persistent neural activity as a key component for mediating WM in range of behavioral tasks—particular tasks involving spatial memory and manipulation of remembered information. However, it is necessary to more thoroughly test for the generality of persistent activity in different subtypes of WM (e.g., verbal, non-spatial). Secondly, the rhythmic model suggests that gamma bursts are coupled with the representation of WM, and beta bursts are associated with the suppression of informative spiking. However, it still lacks evidence of how modulation of different frequencies of WM-related oscillations affects behavioral outputs in a WM task. Moreover, there are conflicting results of what frequency ranges these different phases of oscillations cover across various studies, emphasizing the need for a deeper understanding of the roles of different oscillations in WM, rather than in a specific WM task. Thirdly, the activity silent model proposes that the representation of WM information can be maintained even without elevated populational activity through transient changes in synaptic weights or neuronal excitability, rather than actively maintaining WM information with persistently elevated spiking. While these models may appear to conflict with one-other, they actually provide complementary insights into different aspects of WM function. Recent studies have even shown that both persistent activity and activity-silent dynamics can coexist and switch actively during the same WM task, highlighting the need for a better understanding of how these different dynamics contribute to overall WM functionality (Barbosa et al., 2020; Froudist-Walsh et al., 2021). Lastly, although research into non-invasive stimulation provides promising results for treating WM decline, there is a gap in understanding the mechanism of how stimulation improve WM ability. As for the WM models mentioned above, there is a pressing need to understand how different stimulation contributes to different components of WM function to design optimized stimulation paradigms and even novel approaches to enhance and restore cognitive functioning in patients with WM deficits.

## Author contributions

SL conceived of and organized the article. SL, MR, SC, and SD wrote the initial draft. All authors contributed to the article and approved the submitted version.
